# The Effects of Mindfulness-Based Cognitive Therapy on Affective Memory Recall Dynamics in Depression: A Mechanistic Model of Rumination

**DOI:** 10.3389/fnhum.2012.00257

**Published:** 2012-09-19

**Authors:** Marieke Karlijn van Vugt, Peter Hitchcock, Ben Shahar, Willoughby Britton

**Affiliations:** ^1^Cognitive Modeling Group, Department of Artificial Intelligence, University of GroningenGroningen, Netherlands; ^2^Brown University Contemplative Studies Initiative, Brown UniversityProvidence, RI, USA; ^3^Department of Psychiatry and Human Behavior, Brown University Medical School, Brown UniversityProvidence, RI, USA; ^4^School of Psychology, Interdisciplinary Center HerzliyaHerzliya, Israel

**Keywords:** memory, emotional processing, mindfulness, free recall

## Abstract

**Objectives:** converging research suggests that mindfulness training exerts its therapeutic effects on depression by reducing rumination. Theoretically, rumination is a multifaceted construct that aggregates multiple neurocognitive aspects of depression, including poor executive control, negative and overgeneral memory bias, and persistence or stickiness of negative mind states. Current measures of rumination, most-often self-reports, do not capture these different aspects of ruminative tendencies, and therefore are limited in providing detailed information about the mechanisms of mindfulness. **Methods:** we developed new insight into the potential mechanisms of rumination, based on three model-based metrics of free recall dynamics. These three measures reflect the patterns of memory retrieval of valenced information: the probability of first recall (Pstart) which represents initial affective bias, the probability of staying with the same valence category rather than switching, which indicates strength of positive or negative association networks (Pstay), and probability of stopping (Pstop) or ending recall within a given valence, which indicates persistence or stickiness of a mind state. We investigated the effects of Mindfulness-Based Cognitive Therapy (MBCT; *N* = 29) vs. wait-list control (*N* = 23) on these recall dynamics in a randomized controlled trial in individuals with recurrent depression. Participants completed a standard laboratory stressor, the Trier Social Stress Test, to induce negative mood and activate ruminative tendencies. Following that, participants completed a free recall task consisting of three word lists. This assessment was conducted both before and after treatment or wait-list. **Results:** while MBCT participant’s Pstart remained relatively stable, controls showed multiple indications of depression-related deterioration toward more negative and less positive bias. Following the intervention, MBCT participants decreased in their tendency to sustain trains of negative words and increased their tendency to sustain trains of positive words. Conversely, controls showed the opposite tendency: controls stayed in trains of negative words for longer, and stayed in trains of positive words for less time relative to pre-intervention scores. MBCT participants tended to stop recall less often with negative words, which indicates less persistence or stickiness of negatively valenced mental context. **Conclusion:** MBCT participants showed a decrease in patterns that may perpetuate rumination on all three types of recall dynamics (Pstart, Pstay, and Pstop), compared to controls. MBCT may weaken the strength of self-perpetuating negative associations networks that are responsible for the persistent and “sticky” negative mind states observed in depression, and increase the positive associations that are lacking in depression. This study also offers a novel, objective method of measuring several indices of ruminative tendencies indicative of the underlying mechanisms of rumination.

## Introduction

Major depressive disorder (MDD) is a debilitating mood disorder that affects almost 19 million adults in the USA at any given time (Regier et al., 1998) and almost 20% of the USA population over a lifetime (Blazer et al., 1994; Kessler et al., 2005). MDD is recurrent and progressive, with the likelihood of recurrence exceeding 80% (Judd, 1997; Mueller et al., 1999).

Neurobiological models characterize depression as insufficient top-down modulation by the prefrontal cortex (PFC), a condition known as “hypofrontality” (Drevets, 2001; Davidson et al., 2002; Ochsner et al., 2002; Siegle et al., 2002, 2007b; Ochsner and Gross, 2005; Johnstone et al., 2007). Hypofrontality can result in multiple manifestations of depression, including biological, emotional, and cognitive. On the biological level, a weak PFC results in a hyperactive amygdala (Siegle and Hasselmo, 2002; Siegle et al., 2007b), and an increase in sympathetic hyperarousal and high levels of cortisol (for a review, see Jindal et al., 2003), resulting in damage to the hippocampus (Sheline et al., 1996, 1999; Bremner et al., 2000; Mervaala et al., 2000; Steffens et al., 2011).

Hippocampal deficits are reflected by impaired recall of specific verbal episodic material (i.e., autobiographical memory; Wolkowitz et al., 1990). While on the one hand, high levels of cortisol impair explicit verbal memory (Newcomer et al., 1999) and the specific details of life events, high levels of cortisol also enhance memory for negatively valenced emotional material (Buchanan and Lovallo, 2001). The result is negatively valenced, emotionally thematic, overgeneral “narrative smoothing,” a process by which preserved fragments of memory are unified by a common theme that omits specific non-congruent details (Heuer and Reisberg, 1990; Burke et al., 1992).

*On the cognitive level*, depression and hypofrontality are associated with over general autobiographical memory (Williams, 1996; Williams et al., 2000), all caused by poor prefrontal control over the amygdala (Fales et al., 2008; Beevers et al., 2010; Cisler and Koster, 2010) and impairment of the hippocampus (Payne et al., 2004). Poor prefrontal control is associated with deficits in executive function, especially cognitive set shifting, or the ability to disengage from a particular mind-set (Austin et al., 1999). Impairment in this ability, often called “cognitive control,” is a hallmark of depression vulnerability (for a review, see Hertel, 1997; Gotlib and Joormann, 2010; Joormann and D’Avanzato, 2010). Poor cognitive control commonly manifests as uncontrolled, repetitive or perseverative behavior and thinking (Hertel, 1998; Watkins and Brown, 2002; Watkins and Mason, 2002; Ward et al., 2003; Martin and Tesser, 2006), often referred to as mental “stickiness” (Joormann et al., 2011).

*On the emotional level*, poor prefrontal control and a hyperactive amygdala are associated with biases in attention and memory away from positive stimuli and toward negative stimuli (Burt et al., 1995; Mathews and MacLeod, 2005; Kellough et al., 2008; Disner et al., 2011) and persistent negative affect and high emotional reactivity in response to stress (Baxter et al., 1989; Bench et al., 1993; Mayberg et al., 1999; Davidson et al., 2000; Pine et al., 2001; Clark et al., 2002; Siegle and Hasselmo, 2002; Myin-Germeys et al., 2003; Meyer et al., 2004; Siegle et al., 2007b; Liu et al., 2012).

Importantly the *duration* or persistence of negative affect appears to be more associated with depression risk than negative affect intensity (Cohen et al., 2005; Gillihan et al., 2010). Individuals with longer durations of negative affect following daily life stressors are more likely to develop depressive symptoms than those who recover more quickly (Cohen et al., 2005). As a result, the persistence or “stickiness” (Joormann et al., 2011) of negative affect has become a central target of interventions. While negative affect in depressed individuals is very sticky, they have a reduced capacity to sustain positive emotion (Heller et al., 2009). This leads to an overall deficit in positive affect (Watson and Clark, 1988). Thus, effective treatment strategies for depression should target decreasing negative affective persistence and increasing positive affect persistence.

This specific profile of neuropsychological impairments is referred to as “rumination.” It consists of an excessive, repetitive, and uncontrolled stream of negatively valenced thoughts and memories that are linked to each other by a common theme (often loss, failure, inadequacy, etc.), that is activated by stress or negative affect. Rumination has been consistently linked with risk of developing depression (Lyubomirsky and Nolen-Hoeksema, 1993; Nolen-Hoeksema et al., 1994), or exacerbating current depression (De Raedt and Koster, 2010) and has therefore been a therapeutic target for interventions. While disorder-specific definitions vary (Ehring and Watkins, 2008), their essential features indicate that rumination is a cognitive activity that is (a) repetitive, (b) uncontrolled, and (c) negatively valenced (McLaughlin and Nolen-Hoeksema, 2011).

Rumination scores have been related to overgenerality of autobiographical memories by depressive patients (Heeren and Philippot, 2011), suggesting that the trains of ruminative thoughts are driven by a common unifying theme that reinforces itself by common (negative) content and by deflecting non-congruent (neutral) information (Whitmer and Banich, 2007). Joormann et al. (2011, p. 982) reflect that “ruminators become stuck on recurrent thoughts that revolve around a specific theme and have difficulty flexibly switching to a new train of thought; such perseveration may reflect difficulties manipulating information in working memory.” This cognitive inflexibility may lead people to become stuck in a particular mind-set (Nolen-Hoeksema and Davis, 1999). Recent research in rumination is increasingly investigating the role of prefrontally mediated cognitive control (Demeyer et al., 2012), especially the ability to manipulate emotional information held in working memory (Koster et al., 2011; De Lissnyder et al., 2012).

Given the prominent role of the hypofrontality across psychiatric conditions, many researchers have attempted to use attention training to improve prefrontal control (Wells, 2000; Penades et al., 2006; Siegle et al., 2007a; O’Connell et al., 2008). A large body of research supports the use of meditation-based mental training as a candidate for strengthening or rehabilitating prefrontal attentional control systems. For example, different forms of meditation practice have been associated with better performance on a wide range of prefrontally mediated attention tasks (Valentine and Sweet, 1999; Wenk-Sormaz, 2005; Brefczynski-Lewis et al., 2007; Chan and Woollacott, 2007; Jha et al., 2007; Pagnoni and Cekic, 2007; Slagter et al., 2007; Srinivasan and Baijal, 2007; Tang et al., 2007; Chambers et al., 2008; Bushell, 2009; Lutz et al., 2009; Goldin and Gross, 2010), with increased activity in the (lateral) PFC (Herzog et al., 1990; Jevning et al., 1996; Khushu et al., 2000; Lazar et al., 2000; Baerentsen et al., 2001; Newberg et al., 2001; Ritskes et al., 2003; Brefczynski-Lewis et al., 2007; Creswell et al., 2007; Farb et al., 2007, 2010; Hölzel et al., 2007), larger volumes of frontal gray matter (Lazar et al., 2005; Pagnoni and Cekic, 2007; Hölzel et al., 2008; Luders et al., 2009) and hippocampus (Hölzel et al., 2008; Luders et al., 2009), and greater PFC inhibition of the amygdala (Brefczynski-Lewis et al., 2007; Creswell et al., 2007; Farb et al., 2007; Goldin and Gross, 2010; Hölzel et al., 2010).

In terms of emotions, meditation training techniques have been found to decrease levels of negative affect, anxiety and depression (Kabat-Zinn et al., 1992; Speca et al., 2000; Grossman et al., 2004; Shapiro et al., 2005; Jain et al., 2007; Kenny and Williams, 2007; Kuyken et al., 2008; Witek-Janusek et al., 2008) as well as decreased emotional reactivity (Arch and Craske, 2006, 2010; Ortner et al., 2007; Tang et al., 2007; Proulx, 2008; Brewer et al., 2009; Pace et al., 2009; Raes et al., 2009; Goldin and Gross, 2010).

On the cognitive level, meditation training has also been associated with changes in information processing such as decreases in the overgenerality of memory (Williams et al., 2000; Heeren et al., 2009; Heeren and Philippot, 2011) and negative biases in attention (Garland et al., 2009; Vago and Nakamura, 2011) and memory (Alberts and Thewissen, 2011), as well as increased efficiency of positive information processing (Roberts-Wolfe et al., 2012).

It is therefore not surprising that meditation has been used as a treatment for rumination. Teasdale et al. (1995) theorized that mindfulness interrupts the elaborative processes that fuel rumination and maintain negative mood, a hypothesis that is gaining empirical support (Heeren and Philippot, 2011). Mindfulness meditation (MM) has been associated with reductions in self-reported rumination scores (Ramel et al., 2004; Jain et al., 2007; Kingston et al., 2007; Shahar et al., 2010; Heeren and Philippot, 2011), and this has been proposed as the mechanism through which mindfulness decreases psychological distress (Heeren and Philippot, 2011). Furthermore, self-reported trait mindfulness scores were found to be inversely correlated with self-reported rumination (Mathew et al., 2010).

While rumination nicely aggregates the various cognitive, affective, and neurobiological impairments in depression, the research on meditation and rumination is limited in several ways. First, the most-often used measure of rumination, the Ruminative Response Scale (RRS) of the Response Styles Questionnaire (RSQ; Nolen-Hoeksema and Morrow, 1991) has also been subject to much scrutiny, as has the whole multifaceted construct of rumination (McLaughlin and Nolen-Hoeksema, 2011). Other scales, like the Cambridge-Exeter Rumination Thinking Scale (Barnard et al., 2007a) represent improvements in construct validity, but are still subject to the limitations of all self-report measures. Self-report scales in general are inherently problematic, and prone to demand characteristics. This is especially true in meditation studies where the intervention itself may change how aware participants are of what is happening within their minds. Second, despite the abundance of objective neurobiological and neuropsychological data in the research on meditation, the research related to rumination consists almost entirely of self-report data, and there have been no detailed psychological or neurobiological mechanistic models of how meditation affects rumination. Third, while information processing bias (toward negative and away from positive) has been addressed in meditation research, less emphasis has been put on features of rumination that reflect its horizontal time course: repetition and uncontrollability. The horizontal time course of emotional responses, or “affective chronometry” has received considerable attention (Davidson et al., 2000). Depression and rumination are not problematic simply because they are negative, but because they are persistent, self-perpetuating, and “sticky” or difficult to disengage from Joormann et al. (2011).

Research indicates that it is the increased *duration* of negative affect following a stressor, rather than the intensity, that is associated with depression risk (Cohen et al., 2005; Gillihan et al., 2010). Similarly, a recent study in our lab found that mindfulness training was associated with decreased duration (not amplitude) of negative affect in response to a laboratory stressor, and that this decrease mediated the effects of mindfulness training on depressive symptoms (Britton et al., 2012).

And finally, rumination refers generally to a non-specific kind of “thinking” that includes both feelings and memories, but more recent research has focused on rumination in relation to memory (Williams, 1996; Williams and Swales, 2004; Raes et al., 2006; Moulds et al., 2007; Joormann et al., 2011). Rumination and deficits in episodic memory, including overgenerality, are highly correlated and thought to be part of the same mood-congruent memory process (Watkins and Teasdale, 2001, 2004; Raes et al., 2006; Barnard et al., 2007b; Heeren and Philippot, 2011). For example, Watkins (2002) reflects that “As a depressed person ruminates on unpleasant information, they are engaging in mood-congruent conceptual elaboration. Not only does this make the information more available to both explicit and implicit retrieval, elaborating this information in a mood-congruent fashion should also serve to enhance explicit and implicit retrieval of information related to this material” (Watkins, 2002, p. 398). More recent models have considered rumination to be tightly associated with the ability to manipulate emotional information in working memory (Joormann et al., 2011; Demeyer et al., 2012).

This paper attempts to address some of these gaps by investigating the effects of MM training on an objective neuropsychological measure of memory retrieval. Since rumination is defined as repetitive, uncontrolled, and persistent retrieval of negatively valenced and thematically unified memories, memory retrieval seems to be the central component of this process. Thankfully, detailed computational models exist of memory retrieval that can be used to make specific predictions about the type of retrieval patterns that are associated with rumination. Such retrieval patterns should capture not only overall emotional bias, but also the persistence or “stickiness” of negative mind states that are observed in rumination. Specifically, cognitive inflexibility or stickiness is reflected in long chains of negatively valenced information, whereas cognitive flexibility or cognitive control is reflected in the ability to disengage from negative trains of thought, which can manifest as both shorter negative trains, and/or longer positive ones. In this way we can assess whether mindfulness improves mental stickiness through better cognitive control, and/or a change in the relative strength of negative and positive association networks.

**Figure 1:**
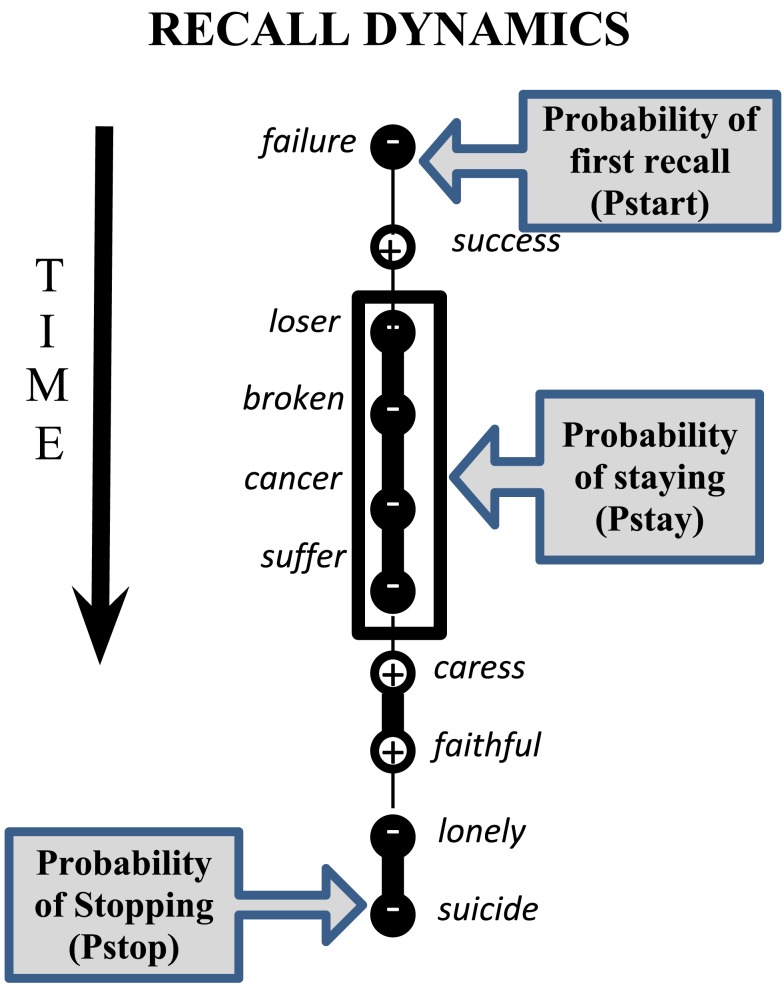
**Schematic overview of recall dynamics**. Words are consecutively recalled are displayed vertically. Negatively valenced words are marked by a black sphere with a “−”, while positively valenced words are marked with a “+”. The vertical lines between valence signs indicates the strength of the association (i.e., heavier lines denote stronger associations).

These concepts can be formalized with “recall dynamics,” which refer to distinct patterns of memory retrieval that have been described by the Temporal Context Model (TCM; Howard and Kahana, 2002) and Context Maintenance and Retrieval (CMR) model (Polyn et al., 2009). During free recall, participants recall words in any order without external cues (Moscovitch, 1994; Stuss et al., 1994; Gershberg and Shimamura, 1995; Becker and Lim, 2003), and the order of recall reveals internally driven organization schemes that drive memory search. Recall dynamics describe how participants proceed from one recall to the next, which is related to how the connection strengths between memories in their neural networks are configured (Kahana, 1996; Nelson et al., 2004; Tse, 2009). Organization of recall provides a window into the way people search through their memory and construct trains of thought (e.g., Howard and Kahana, 2002; Kahana and Miller, 2011). The order of recall is driven by an internally maintained contextual or preexisting semantic or thematic commonality among to-be-remembered items. For example, words that are unified by a common theme, such as negative valence, will be strongly associated with each other and therefore recalled consecutively (e.g., “failure,” “loser” “misery” “sad” – see Figure 1), a phenomenon known as semantic clustering (Kahana and Miller, 2011). The length of the train of negative words can yield information about the strength of the negative association network. The length of valence trains and pattern of transitions between positive, negative, and neutral words also reflects the strength of prefrontal control. This is because the prefrontal cortex plays a crucial role in recall dynamics (Becker and Lim, 2003) as it controls the internal representations that organize recall (Polyn and Kahana, 2008) and is also responsible for switching to new organization schemes (Rudy et al., 2005).

In this study, we used three types of recall dynamics (probability of first recall, recall transitions, and probability of stopping, Pstop; Kahana, 2012) to describe the potential mechanisms underlying rumination. To be able to do this, we adapted their definitions to focus on the valence of the words, rather than their serial position, which is usually considered. The “probability of first recall” (Pstart; Hogan, 1975) then describes the valence of words at the beginning of recall which gives a window into the initial affective state of a given participant (Tulving and Donaldson, 1972). Participants start recall by retrieving an item consistent with their current mental state (Howard and Kahana, 2002). As such, a participant experiencing a negative affective state is more likely to remember a negative word from the list. If mindfulness training decreases overall negative bias in response to a stressor, we expect to see decreased probability of recalling a negatively valenced word as the first word after experiencing this stressor.

“Recall transitions” describe how often people transition from one category of word to another (Howard and Kahana, 1999; Kahana, 2000). In the context of affective word lists, this would mean counting how often people make transitions from one valence category to another (similar to Polyn et al., 2009 who used this method for non-affective categories). Long trains of negative words indicate a strong network of negative associations, while shorter negative trains indicate both weaker associations and greater ability to disengage from negative information (i.e., prefrontal control). If mindfulness training decreases the strength of negatively valenced associations, or improves set shifting ability, we expect mindfulness-trained participants to stay with trains of negative words for shorter periods.

“Pstop” is the probability that a given item is the last item recalled, and it therefore increases with each subsequent item recalled. Pstop thus differs from Pstart in that it measures what the mental context has drifted toward when memories have been exhausted, rather than the mental context from which recall starts. If mindfulness decreases the persistence or stickiness of negative mental contexts, we expect mindfulness-trained participants decrease in their probability of ending recall with a negative valence word (Forgas and Bower, 1987).

We tested these hypotheses in a sample of partially remitted depressed patients who were randomized to either 8 weeks of mindfulness-based cognitive therapy (MBCT) or a wait-list control. Following a standard laboratory stressor, the Trier Social Stress Test (TSST; Kirschbaum et al., 1993) as a negative mood induction to activate ruminative tendencies (Nolen-Hoeksema et al., 2008), participants completed a free recall task consisting of three word lists, both before and after treatment or wait-list.

Specifically, we hypothesized that after the intervention: (1) MBCT-trained participants are less likely than controls to start off recall with a negatively valenced word (2) MBCT-trained participants persist in recalling trains of positively valenced words for longer periods, and in recalling trains of negatively valenced words for shorter periods, reflecting decreased strength of negative associations, and increased strength of positive associations within the MBCT group compared to controls. (3) MBCT-trained participants are less likely to finish their recall with a negative word, reflecting a decreased tendency of this group to get stuck in negative mind states.

## Materials and Methods

### Participants

Participants (*N* = 52) were individuals with a recurrent form of unipolar depression in partial or full remission with varying degrees of residual symptoms (see Britton et al., 2012, for details). Structured Clinical Exclusion criteria included (a) a history of bipolar disorder, cyclothymia, schizophrenia, or other psychotic disorders, persistent antisocial behavior or repeated self-harm, borderline personality disorder, organic brain damage, (b) current panic, obsessive-compulsive disorder, eating disorder, or substance abuse/dependence, (c) inability to read/write in English, (d) current psychotherapy, or (e) a regular meditation practice. Individuals on antidepressants were permitted to participate as long as they reported no change in medication type or dose during the 3 months prior to enrollment or during the active phase of the study.

### Self-report measures

*Depressive symptoms* were measured by the Beck Depression Inventory (BDI; Beck and Steer, 1987). The BDI is a widely used measure of depressive symptoms and has excellent psychometric properties (Beck et al., 1988). The BDI had an internal consistency coefficient of 0.81 before treatment and 0.90 at post-treatment.

#### Rumination

The 22-item RRS of the RSQ (Nolen-Hoeksema and Morrow, 1991) was used to measure changes in self-reported rumination. In this sample, the internal consistency coefficient was 0.88 at both pre- and post-treatment.

#### Anxiety and negative affect

The Spielberger State-Trait Anxiety Inventory form Y (STAI-Y1; Spielberger et al., 1983) is a 20-item self-report inventory where respondents rate their current levels of negative affect on a four-point Likert scale. In the current sample, internal consistency ranged from 0.89 to 0.93. The STAI was completed before and after the TSST (see Britton et al., 2012). In this report, the STAI is used to demonstrate the efficacy of our negative mood induction procedure.

### Laboratory based measures

#### Trier social stress test

Before doing the recall task, participants underwent a TSST. The TSST is a procedure that reliably produces moderate psychological distress in laboratory settings (Kirschbaum et al., 1993). A full description of the TSST can be found in Britton et al. (2012). Briefly, subjects prepared and delivered a speech, followed by an oral subtraction task in front of a panel of judges, stage lights, and cameras. We used the TSST to induce self-evaluation and negative affect that would make rumination more probable and enduring and to magnify the recall bias of depressive patients toward negative words (Nolen-Hoeksema et al., 2008). This negative recall bias is expected because TSST causes an increase in stress (Kirschbaum et al., 1992; Williams et al., 2004), and an increase in stress has be shown to cause impairments in the recall of positive and neutral words even for non-depressed individuals (Kirschbaum et al., 1996; Tops et al., 2003), and increase in recall of negative valence words (Abercrombie et al., 2003) and in rumination (e.g., Roger and Najarian, 1998).

#### Free recall task

Participants were presented with three lists of 22 words, six positive (normative valence = 7.5 ± 0.40, arousal = 5.8 ± 0.80), six negative (valence = 2.6 ± 0.73, arousal = 5.5 ± 1.37), and six neutral words (valence = 5.18 ± 0.34, arousal = 3.84 ± 0.48). Four neutral buffer words appeared at the beginning and end of each list to control for primacy and recency effects and were excluded from the analysis. Words were block-randomized with no words of the same valence appearing consecutively. Lists were balanced for valence, arousal, length, and frequency of appearance in the English language. Based on the suggestions of Koster et al. (2010) to assess multiple modalities in the same subject to confirm that the findings are not limited to any one form of processing, subjects received two visually presented lists separated by an aurally presented list. Visual lists were presented in white font on a black or red background, and aurally presented lists were delivered at a volume level set by participants. Stimuli were presented on a 15-inch computer screen using DMDX software (Forster and Forster, 2003). Words were shown for 6 s each, followed by a 500-ms blank screen. Two separate sets of lists were counterbalanced for pre- and post-treatment administration. Participants rated each word (1–9) on the dimensions of pleasantness and arousal to ensure depth of encoding. Immediately after this encoding phase, participants were asked to recall these words in any order in an incidental memory test. Recall did not differ between visual vs. aural presentation so all three lists are collapsed for the analyses to maximize statistical power. Time is coded as T1 (before the MBCT program or wait-list) and T2 (after the MBCT program or wait-list).

### Procedure

After completion of baseline assessments, participants were block-randomized (block size = 5) to a MBCT program or wait-list control condition in a 3:2 ratio without reference (stratification) to baseline characteristics, using identical, shuffled, opaque, sealed envelopes (Doig and Simpson, 2005). After 8 weeks of treatment or wait-list condition, participants completed a post-treatment questionnaire packet and returned to the laboratory for assessment. All self-report measures and tasks were administered by personnel that was blinded to the treatment condition (MBCT vs. control) of the participants (see Britton et al., 2012, for details). The experiments were conducted between May 2004 and December 2005 at the University of Arizona, Department of Psychology in Tucson, Arizona. The study protocol was approved by the University of Arizona Institutional Review Board, and all participants provided written informed consent. No adverse events occurred during the trial.

### Intervention – mindfulness-based cognitive therapy

Mindfulness-Based Cognitive Therapy (Segal et al., 2002, 2004; Teasdale, 2004) is an 8-week group-based intervention that combines principles from cognitive-behavioral therapy (Beck et al., 1979) and Mindfulness-Based Stress Reduction (MBSR; Kabat-Zinn, 1990) using a psycho-educational and client-centered format. MBCT sessions focus on cultivating mindfulness or non-judgmental present-moment awareness of mental content and everyday activities. Homework assignments consist of practicing MM exercises with the aid of a guided audio CD. A session-by-session description with handouts and homework assignments is available in the MBCT manual (Segal et al., 2002). Sessions were conducted by the last author who had more than 10 years of mindfulness practice experience and has received extensive training in delivery of the program through the Center for Mindfulness MBSR Instructor Certification Program at University of Massachusetts Medical School, and through MBCT training with Dr. Zindel Segal, the first author of the MBCT manual. According to Segal et al. (2002), mindfulness training breaks the high level thematically driven elaboration of repetitive negative thoughts by redirecting focus to a lower level, concrete focus (body sensations).

#### Analysis methods

The self-report measures, as well as the mean number of recalls, were analyzed with standard repeated measures analysis of variance (ANOVA), with factors treatment group (MBCT vs. control), and time (before vs. after the MBCT program). Because of the limited number of lists per participant, and the inappropriateness of ANOVAs for probabilities (Jaeger, 2008) we used non-parametric permutation analogs of ANOVAs for assessing free recall dynamics. Because outliers have very little effect on non-parametric permutation tests, we did not remove any outliers from the data.

We examined three measures of free recall dynamics: the probability of first recall (Pstart), Pstop, and probability of staying rather than switching (Kahana, 2012), which we adapted to study the effects of valence on recall dynamics. Because of the limited number of lists per participant, we combined the data from all participants. Pstart is defined in our design with three affective categories as the probability that a participant starts recall with an item from that valence category. Similarly, Pstop is the probability that a participant will stop after recalling an item of a particular affective category. Response transitions were analyzed in terms of the probability of continuing recalling within a specific valence category (i.e., staying rather than switching).

For every recall dynamics measure (i.e., probability of first recall, probability of staying, Pstop), we determined *p*-values with a permutation procedure (Wasserman, 2004). In this procedure, the labels of treatment and time point were randomly swapped, and the probabilities were recomputed, for 1000 iterations (changing the number of iterations did not affect our results). This created a permutation distribution for the null hypothesis of no effect of treatment and time point. The *p*-value was the probability of the empirical recall dynamics measure compared to the permutation distribution of recall dynamics measures. Permutation analogs of ANOVA were implemented by subtracting the conditions of interest, repeating the subtraction for the randomized data, and computing where the empirical recall dynamics measure fell in the corresponding permutation distribution (Hesterberg et al., 2005). These statistical tests were implemented in Matlab (Mathworks, Inc., Natick, MA, USA).

## Results

### Participant flow and compliance

Of the 52 participants that completed baseline assessments, 23 were randomized to wait-list control and 29 to MBCT. Twenty-six (90%) MBCT and 19 (82%) wait-listed participants completed all assessments (total *N* = 45). Outside of class, the 26 completers reported engaging in formal MM practice an average of 39.94 ± 10 min/day, 5.2 ± 1.2 days/week. According to the goal of 45 min/day, 6 days/week of formal MM practice (270 min/week = 100%), the average number meditation minutes across all weeks was 76 ± 24% with a range of 79–308 min/week.

### Manipulation check: TSST reliably induces negative affect

We conducted a series of analyses to evaluate the effectiveness of the TSST in producing negative affect/anxiety, and to assess whether repeated administration led to an attenuated response (i.e., habituation). Using the change between baseline and the report of anxiety during the speech, the TSST produced a significant increase in anxiety for all participants at both pre- [*t*(44) = 7.5, *p* < 0.001 $\eta_{p}^{2}$ = 0.55] and post-treatment [*t*(44) = 5.9, *p* < 0.001, $\eta_{p}^{2}$ = 0.44]. There was no attenuation in the peak level of anxiety produced by the TSST from pre- to post-treatment assessment [STAI score during speech at pre-treatment = 53.4 ± 10.9, at post-treatment = 50.7 ± 11.12, main effect of time, *F*(1, 42) = 2.3, *p* = 0.14].

### Self-report measures

Baseline scores for all these measures are reported in Table 1.

**Table 1:** **Baseline measures**.

	Mean (SD) T1		Minimum T1		Maximum T1	
Age	46.6 (7.8)	47.1 (12.3)	25	24	59	64
%Female	76.9 (43.0)	93.8 (25.0)	–	–	–	–
BDI	9.1 (6.1)	9.0 (5.7)	0	2	21	21
RRS	51.3 (9.7)	47.9 (11.9)	35	34	67	68
STAI	40.0 (11.7)	40.7 (9.3)	20	27	62	63

*SES, socio-economic status; BDI, Beck Depression Inventory; RRS, rumination response scale; STAI, Spielberger Trait Anxiety Questionnaire*.

#### Depression

As expected, MBCT treatment was successful; we found a significant decrease in BDI over time [*M*_time1_ = 9.1, *M*_time2_ = 6.5, *F*(1, 42) = 6.6, *p* < 0.05, $\eta_{p}^{2}$ = 0.14], with a trend toward a significant effect of group [*F*(1, 42) = 3.2, *p* = 0.08, $\eta_{p}^{2}$ = 0.07] and a significant interaction between time and group [*F*(1, 42) = 6.0, *p* < 0.05, $\eta_{p}^{2}$ = 0.13]. The interaction indicates that the treatment effect is larger for the MBCT group than for the control group.

#### Rumination

There was a significant group × time interaction [*F*(1, 42) = 13.3, *p* < 0.001, $\eta_{p}^{2}$ = 0.24], suggesting a pattern of decreased self-reported rumination for the MBCT group in comparison to controls.

#### STAI anxiety

As previously reported in Britton et al. (2012), a significant group x time interaction indicated that TSST-related anxiety decreased more than controls in the MBCT group [*F*(1, 42) = 6.2, *p* < 0.05, $\eta_{p}^{2}$ = 0.13].

### Word recall

#### Overall recall

Participants recalled on average 43.6% of the words at T1 and 48.4% at T2. Recall accuracy improved from T1 to T2 [*F*(1, 42) = 547.5, *p* < 0.001, $\eta_{p}^{2}$ = 0.93] but did not differ by group.

We then compared the average recall probabilities for the three different valence categories (Table 2). Participants recalled more positive, negative, and neutral words at T2 than at T1 [*F*(1, 42) > 236, *p* < 0.001, $\eta_{p}^{2}$ > 0.85], indicating they improved on the task through practice. There were no main effects of treatment or interactions between time and treatment on the average number of words recalled.

**Table 2:** **Mean percentage of recalled words by valence category, treatment group, and time point**.

Group	T1	T2
**POSITIVE VALENCE**		
MBCT	28.9%	33.5%
Control	27.3%	30.5%
**NEGATIVE VALENCE**		
MBCT	27.2%	27.4%
Control	24.0%	32.0%

### Recall dynamics

#### Probability of first recall

**Figure 2:**
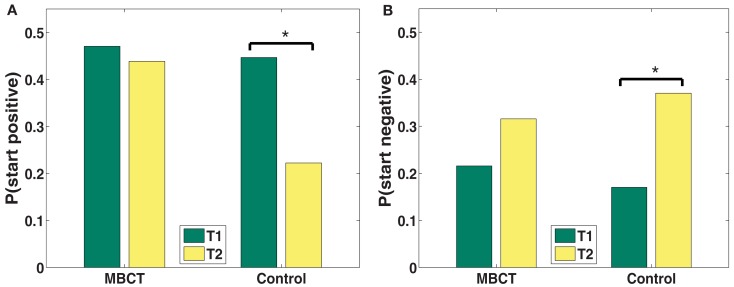
**(A,B)** Changes in probability of first recall from T1 to T2 by valence category (**A**: positive; **B**: negative). *p < 0.05.

The probability of first recall (Pstart) gives a window into what the mental context is of the participant (Tulving and Donaldson, 1972) at the time of the start of recall. People start recall by cuing with the current mental context (Howard and Kahana, 2002), which results in the retrieval of items that share elements with this contextual state. For an affectively negative mental context, negative valence items will be preferentially retrieved from memory. While MBCT’s Pstart remained relatively stable from T1 to T2, controls showed multiple indications of deterioration (Figure 2). Specifically, controls showed an increased probability of starting recall with a negative word (*p* < 0.05; see also Table 3) and a decreased probability of starting with a positive word (*p* < 0.05).

**Table 3:** Statistics for permutation tests of changes in recall dynamics between T1 and T2 (Pstart), for the two groups, and for the interaction between group and time (**p < 0.001; *p < 0.05;^†^ p < 0.1).

**Pstart**	**Negative valence**	**Positive valence**
Control	*p* = 0.039*	*p* = 0.037*
MBCT	*p* = 0.126	*p* = 0.335
Interaction	*p* = 0.224	*p* = 0.123
**PSTAY**		
Control	*p* < 0.001**	*p* < 0.001**
MBCT	*p* < 0.001**	*p* < 0.001**
Interaction	*p* < 0.001**	*p* < 0.001**
**PSTOP**		
Control	*p* = 0.237	*p* = 0.108
MBCT	p = 0.047*	*p* = 0.070^†^
Interaction	*p* = 0.067^†^	*p* = 0.496

#### Recall transitions

**Figure 3:**
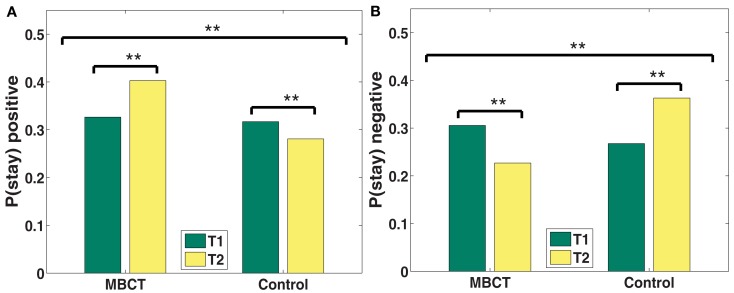
**(A,B)** Changes in probability of staying within the same category from T1 to T2 by valence category (**A**: positive; **B**: negative). *p < 0.05, **p < 0.001.

We then asked how participants transition between list items. In essence, recall transitions say something about how participants’ trains of thoughts develop over the course of recall, and in the case of affective words, about the strength of associations between words of a specific valence category. Transitions can be quantified with the probability of persisting in recalling words of a particular valence category, rather than switching to a different valence category. Significant group-by-time interactions indicate that MBCT participants increased more than controls in their tendency to make transitions from positive to positive words, and decreased more than controls in their tendency to make negative to negative transitions (Figure 3). From T1 to T2, positive associations became increasingly more available for recall MBCT participants (*p* < 0.001; see also Table 3), but less available for recall for controls (*p* < 0.001). Conversely, the strength of negative associations decreased for the MBCT group (*p* < 0.001), but increased for the control group.

#### Probability of stopping

Whereas Pstart reflects the participant’s mental context at the start of the recall episode, Pstop indexes the mental context toward which the participant drifts over the course of the recall process (Sederberg et al., 2008) and thus is an indicator of the persistence or stickiness of that context or mental state. A strong network of negative associations will reinforce itself, and make it more difficult retrieve positive words (Forgas and Bower, 1987).

**Figure 4:**
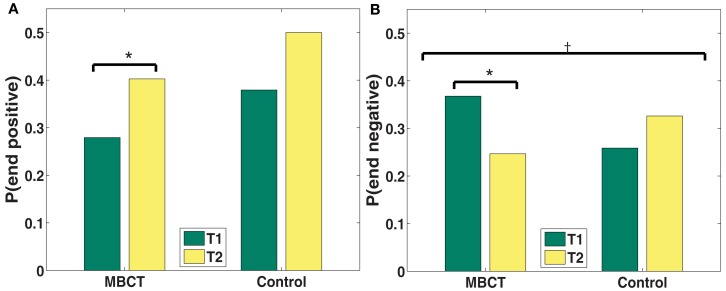
**(A,B)** Changes in probability of stopping from T1 to T2 by valence category (**A**: positive; **B**: negative). *p < 0.05, ^†^*p* < 0.1.

A trend toward a significant group and time interaction (*p* = 0.07; see also Table 3) for negative words (see Figure 4) indicates that the MBCT group decreased more than the control group in their tendency to end recall with a negative word. Indeed, the probability of ending with a negative word decreased significantly for the MBCT (*p* < 0.05), but increased for controls. The probability of ending on a positive word increased at the trend level (*p* = 0.07) for MBCTs but not for controls.

## General Discussion

We examined for the first time model-based measures of recall dynamics that could give insights into the mechanisms underlying rumination. We investigated the effects of MBCT vs. wait-list control on these recall dynamics in a RCT in individuals with recurrent depression. We found that all three types of recall dynamics (probability of first recall, recall transitions, and Pstop) indicate an improvement in tendencies conducive to rumination following MBCT compared to controls. We discuss the results of each measure of recall dynamics below.

### Probability of first recall

The valence of the first words recalled indicates the initial affective bias of the mind state that results from having just experienced the TSST. While MBCT’s Pstart remained relatively stable, controls showed multiple indications of depression-related deterioration toward more negative and less positive bias.

### Recall transitions

The likelihood of staying or switching out of a given valence category indicates the strength of associations within that category. For example, long trains of negative words indicate a strong negative network of negative associations, whereas a short train indicates the tendency to switch to another (non-negative) category. Following the intervention, MBCT participants decreased in their tendency to sustain trains of negative words and increased their tendency to sustain trains of positive words. Conversely, controls showed the opposite tendency: controls stayed in trains of negative words for longer, and stayed in trains of positive words for less time relative to at T1. This indicates that negative associations were less available and positive associations were more available to MBCT participants after their training.

### Probability of ending recall

While the valence of the first words recalled indicate initial affective bias, the valence of words recalled at the end of recall indicate the contextual drift of a mind state. In comparison to controls, MBCT participants tended to stop recall less often with negative words. Decreased stopping with negative words indicates that MBCT participants are less likely to drift toward or persist in a negatively valenced mental context.

There is accumulating evidence that mindfulness training exerts its therapeutic effects by decreasing rumination (Ramel et al., 2004; Jain et al., 2007; Kingston et al., 2007; Mathew et al., 2010; Shahar et al., 2010; Heeren and Philippot, 2011). Specifically, the creators of MBCT theorized that mindfulness training breaks the high level thematically driven elaboration of repetitive negative thoughts by redirecting focus to a lower level, concrete focus such as body sensations (Teasdale et al., 1995; Segal et al., 2002). Our results give experimental support for the suggestion that MBCT trains the ability to disengage from self-perpetuating negative associations held in memory.

While the theorized mechanism of MBCT includes the improved ability to disengage and thereby weaken negative associations, there is no theory supporting an increase in the strength of positive associations. Another study from our lab demonstrated that mindfulness training is associated with an increased efficiency in positive memory recall, which was correlated with improvements in anxiety, depression, and overall well-being (Roberts-Wolfe et al., 2012). Since depression is characterized not only by the persistence of negative affect but also the paucity of positive affect and inability to sustain it (Watson and Clark, 1988; Heller et al., 2009), this finding is of high clinical relevance.

On a neural level, the tendency toward excessive, repetitive, and uncontrolled negative thoughts and memories that are linked to each other by a common theme, often called rumination but also characteristic of overgeneral memory, is caused by the combination of a weak prefrontal cortex and consequently dysregulated hippocampus and amygdala. The ability to shift mental sets or disengage from negative emotional information held in memory is associated with prefrontal control, also called cognitive flexibility. Our findings are consistent with the idea that mental training increases the strength of prefrontal attention and executive brain networks, with improved modulation of the hippocampus and amygdala (Brefczynski-Lewis et al., 2007; Creswell et al., 2007; Farb et al., 2007; Goldin and Gross, 2010; Hölzel et al., 2010; see Hölzel et al., 2011, for a review).

Our findings are also consistent with models that describe depression as a progressive sensitization to stress (Segal et al., 1996, 1999, 2006). In an investigation of affective response to the TSST (Britton et al., 2012), we found that while the MBCT group recovered faster from stress, controls showed evidence of stress sensitization. They returned to the lab for the second TSST with higher levels of pre-speech anticipatory anxiety than the first TSST. The pattern of recall dynamics followed a similar pattern: after an 8-week wait-list, controls were more likely to start with a negative word and less likely to start with a positive word. Controls were also more likely to have long trains of negative associations and less likely to have positive trains. Together, these data suggest that recall dynamics not only can measure the effects of interventions, but also the progressive vulnerability that puts individuals at risk for recurrence.

While the current study confirmed our hypotheses about the effect of mindfulness training on recall dynamics, it also yielded several other findings that are worth mentioning. First, there has been much speculation that meditation may improve cognitive abilities, including memory, but our findings do not support this idea: MBCT training did not improve overall memory ability (total words recalled) more than the control condition. In addition, MBCT did not influence overall affective memory bias, which suggests that using total word counts is not a very sensitive measure of bias.

### Strengths and limitations

This study represents an attempt to develop a biologically informed objective measure of rumination based on validated computational models. Recall dynamics may offer a more objective measure than self-reports and a more nuanced and sensitive measure of affective memory biases that includes the quality of stickiness or persistence. In addition to this strength, the study has many limitations. First, our study used many fewer word lists than typical recall dynamics studies (see Howard and Kahana, 1999), which limits our reliability and statistical power. Future studies should use 25 or more lists per participant in order to permit the use of individual level statistics that assess relationships between self-reported rumination and recall dynamics.

Second, because the MBSR instructor was also the principal investigator, she was not blind to the hypothesis and could influence results by “teaching to the test.” However, in comparison to both subjective reports and word-count based affective bias measures, recall dynamics are probably more robust in the face of demand characteristics or experimenter effects.

Finally, while we found group-level difference in recall dynamics, this study says nothing about mechanisms of action or which component of MBCT were responsible for the differences. While we report in terms of the effects of “mindfulness,” the cognitive therapy component is also a likely candidate for changing patterns of recall associations because its basis is gaining awareness of thought patterns and changing them.

Although the behavioral results we demonstrate here have weak statistical power, and should be replicated by other experiments, we suggest that the methods developed here for analyzing patterns of associations between words of different valences could be used to address many questions in the domain of affective functioning and memory. They provide an important new perspective on the study of the emotion-cognition interface, particularly as it is related to mindfulness.

Using novel methods for quantifying the memory dynamics that may underlie rumination, we investigated patterns of memory recall dynamics before and after MBCT or a wait-list control. Analysis of recall dynamics suggest that MBCT may weaken the strength of self-perpetuating negative associations networks that are responsible for the persistent and “sticky” negative mind states observed in depression, and increase positive associations that are lacking in depression. This study also offers a novel biologically informed, objective method of measuring several indices of ruminative tendencies.

## Conflict of Interest Statement

The authors declare that the research was conducted in the absence of any commercial or financial relationships that could be construed as a potential conflict of interest.
